# Disitamab vedotin plus toripalimab as a first-line treatment for HER2-expressing advanced urothelial cancer: a cost-effectiveness analysis from China based on the RC48-C016 trial

**DOI:** 10.3389/fphar.2026.1824661

**Published:** 2026-07-17

**Authors:** Xiaolin Yun, Qiongshi Wu, Chenxin Zeng

**Affiliations:** 1 Department of Pharmacy, Hainan General Hospital, Haikou, China; 2 Department of Pharmacy, Jinhua Municipal Central Hospital, Jinhua, China

**Keywords:** advanced urothelial cancer, chemotherapy, cost-effectiveness, disitamab vedotin, partitioned survival model, toripalimab

## Abstract

**Background:**

The phase III RC48-C016 trial demonstrated that disitamab vedotin plus toripalimab (DV + T) provides significant survival benefits compared to chemotherapy in patients with HER2-expressing advanced urothelial cancer (UC), along with a favorable safety profile. However, its cost-effectiveness has not yet been evaluated. This study aims to evaluate the cost-effectiveness of DV + T versus chemotherapy as first-line treatment for HER2-expressing advanced UC from the perspective of the Chinese healthcare system.

**Methods:**

A partitioned survival model with a 15-year time horizon was developed to evaluate the cost-effectiveness of DV + T compared to chemotherapy. Outcomes included total cost, quality-adjusted life year (QALY), and the incremental cost-effectiveness ratio (ICER). Model uncertainty was examined through one-way and probabilistic sensitivity analyses.

**Results:**

DV + T yielded 2.44 QALYs at a total cost of $98,514.95, while chemotherapy yielded 1.43 QALYs at a cost of $28,300.87. The ICER was $69,575.31 per QALY. In China, at a willingness-to-pay (WTP) threshold of $20,167.02 per QALY, DV + T has a 0% probability of being cost-effective as a first-line treatment for HER2-expressing advanced UC compared to chemotherapy. Subgroup analysis suggested that the ICER of DV + T versus chemotherapy tended to be substantially lower in patients ineligible to receive cisplatin ($61,264.33 per QALY) than in those eligible for cisplatin-based chemotherapy ($245,983.12 per QALY).

**Conclusion:**

From the perspective of the Chinese healthcare system, DV + T is not cost-effective as first-line treatment for HER2-expressing advanced UC compared to chemotherapy.

## Introduction

Urothelial cancer (UC), which constitutes about 90% of all bladder cancers, is a major global health burden and the ninth most common malignancy worldwide (Nadal and Bellmunt, 2019; Bray et al., 2024). Approximately 5% of patients present with distant metastasis at diagnosis, and about 50% experience recurrence within 2 years after surgery (Patel et al., 2020). Patients with advanced UC have a poor prognosis, with a five-year survival rate below 10% (National Institutes of Health and National Cancer Institute). Platinum-based chemotherapy has been the cornerstone of first-line treatment for decades, however, its efficacy remains limited, with patients achieving a median progression-free survival (PFS) of 7–9 months and a median overall survival (OS) of approximately 16–18 months (Kawa et al., 2024; von der Maase et al., 2005). While immune checkpoint inhibitors (ICIs) have improved outcomes for a subset of patients, the overall clinical benefit remains constrained, underscoring a critical need for more effective therapeutic strategies (Balar et al., 2017; Galsky et al., 2020). Therefore, it is imperative to identify new therapeutic targets in advanced UC.

Human epidermal growth factor receptor 2 (HER2) is highly prevalent in UC beyond breast cancer, with 52%–69.8% of persons with UC having a score of at least 1+ on HER2 immunohistochemical (IHC) assay, presenting a viable therapeutic target (Koshkin et al., 2025; Zhou et al., 2023; Uzunparmak et al., 2023). Disitamab vedotin is a novel HER2-targeting antibody-drug conjugate that delivers the cytotoxic payload monomethyl auristatin E (MMAE) directly to tumor cells, inhibiting mitosis and inducing apoptosis (DeeksVedotin, 2021). Toripalimab is a humanized PD-1 monoclonal antibody that reinvigorates antitumor immunity (Keam, 2019). RC48-C016, a phase III trial, was the first to evaluate the DV + T regimen as a first-line treatment in Chinese patients with HER2-expressing advanced UC. The combination significantly improved both OS and PFS, with a median OS of 31.5 months versus 16.9 months (hazard ratio [HR], 0.54; 95% CI, 0.41 to 0.73; P < 0.001) and a median PFS of 13.1 months versus 6.5 months (HR, 0.36; 95% CI, 0.28 to 0.46; P < 0.001) (Sheng et al., 2025).

Despite its clinical benefits, the substantial increase in healthcare costs associated with DV + T cannot be overlooked, particularly in resource-constrained settings like China. Therefore, a comprehensive economic evaluation is necessary to assess its cost-effectiveness as a first-line treatment for advanced UC, balancing clinical value against financial impact. Currently, no such study has been conducted in the Chinese context. To address this evidence gap, this study conducts an economic evaluation to assess the cost-effectiveness of DV + T versus chemotherapy in the context of the Chinese healthcare system, utilizing clinical data from the RC48-C016 trial.

## Methods

### Clinical information

The clinical data used in this study were obtained from the RC48-C016 trial. Eligible patients were at least 18 years of age; had histopathologically confirmed, unresectable, locally advanced or metastatic urothelial cancer for which they had not previously received systemic chemotherapy; and had no disease progression or recurrence within 12 months after receiving neoadjuvant or adjuvant treatment. Patients had to have a centrally confirmed HER2-expressing tumor (IHC score of 1+, 2+, or 3+), Patients assigned to the DV + T group received DV at a dose of 2 mg/kg of body weight and T at a dose of 3 mg/kg once every 2 weeks. Patients assigned to the chemotherapy group received gemcitabine at a dose of 1,000 mg/m^2^ on days 1 and 8 of each 3-week cycle plus either cisplatin at a dose of 70 mg/m^2^ or carboplatin at a dose of 4.5 mL/min (as calculated with the use of the Calvert formula) intravenously on day 1 of each 3-week cycle. The proportion of patients receiving carboplatin was 46.9%, and those receiving cisplatin was 53.1%. Treatment was continued until disease progression, development of unacceptable toxic effects, or completion of the maximum number of treatment cycles (chemotherapy, six cycles; disitamab vedotin plus toripalimab, no maximum). As the trial did not report comprehensive treatment data after disease progression, our economic model assumed that all patients received best supportive care (BSC) following disease progression or unacceptable toxicity.

### Constructing the model

**FIGURE 1:**
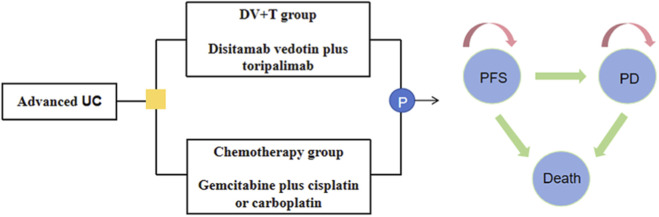
The partitioned survival model simulating outcomes for the RC48-C016 trial. All patients started with PFS state and received treatment with DV + T or chemotherapy. DV + T, disitamab vedotin plus toripalimab; PFS, progression-free survival; PD, progressive disease.

The reporting of this health economic evaluation was conducted in accordance with the Consolidated Health Economic Evaluation Reporting Standards 2022 (CHEERS 2022) statement (Husereau et al., 2022) (Supplementary Table S1). A partitioned survival model was developed from the perspective of the Chinese healthcare system to estimate the costs and health outcomes of DV + T versus chemotherapy. The model comprised three mutually exclusive health states: PFS, progressive disease (PD) and death. All patients entering in the PFS state. Individuals either remained in their current health state or progressed to a different one throughout the simulation, with no option to regress to the previous state. The death state included all-cause mortality (Figure 1). To ensure model credibility, two clinicians assessed face validity, and internal validity was confirmed via extreme-value sensitivity testing and rigorous code verification.

The Kaplan-Meier curves for PFS and OS, as reported in the RC48-C016 trial, were digitized using WebPlotDigitizer (https://automeris.io) to extract individual time-to-event data points. Subsequently, the reconstructed survival data were fitted to a range of standard parametric survival distributions using R (version 4.4.2). The candidate models included the exponential, Weibull, gamma, log-logistic, log-normal, and gompertz, generalized gamma distributions (Supplementary Table S2). The parameters of each candidate distribution were estimated using the maximum likelihood method. Subsequently, models were ranked by trading off goodness-of-fit against complexity according to the Akaike information criterion (AIC) and Bayesian information criterion (BIC), where lower values indicate a better fit (Williams et al., 2017). The optimal models and estimated parameters for each model are summarized in Table 1.

**TABLE 1:** Relevant parameters of survival distributions.

Parameter	Distribution	Value	References
Survival model for DV + T			
OS	log-logistic	Shape = 1.647, Scale = 27.069	Sheng et al. (2025)
PFS	log-logistic	Shape = 1.587, Scale = 13.087	Sheng et al. (2025)
Survival model for chemotherapy			
OS	log-logistic	Shape = 1.599, Scale = 17.608	Sheng et al. (2025)
PFS	Weibull	Shape = 1.445, Scale = 8.329	Sheng et al. (2025)

DV + T, disitamab vedotin plus toripalimab; OS, overall survival; PFS, progression-free survival.

A cycle length of 3 weeks and a time horizon of 15 years were adopted. The WTP threshold was set at 1.5 times China’s 2024 *per capita* GDP ($20,167.02) (Pichon-Riviere et al., 2023; Cai D. et al., 2022). According to the Chinese Pharmacoeconomic Evaluation Guide 2025, an annual discount rate of 4.5% was applied for the costs and utility values. The primary economic outcome was the ICER. A treatment regimen was considered cost-effective if its ICER was below this pre-determined WTP threshold.

### Costs and utility

This study considered direct medical costs only, including drugs, tests, routine follow-up, BSC, terminal care, and management of grade ≥3 adverse events with an incidence >5% (Sheng et al., 2025). Terminal care costs were modeled as a one-time cost incurred in the cycle prior to death. Drug prices were sourced from Yaozhi.net on 10 December 2025, with the prices of gemcitabine, carboplatin, and cisplatin based on national tender data (Yaozh, 2025). Other costs were derived from literature or expert opinion and inflated to 2024 values using the Chinese Medical Care Consumer Price Index. All costs are reported in US dollars (1 USD = 7.1217 CNY). Health-state utilities for PFS and PD states were derived from published literature (You et al., 2024). These utilities, scaled from 0 (representing death) to 1 (representing perfect health), reflect the quality-of-life weights associated with each health state. AE-related disutility values in the first cycle were extracted from other studies and used for the present analyses. Relevant parameters are shown in Table 2.

**TABLE 2:** Model parameters.

Variable	Base value	Minimum	Maximum	Distribution	References
Drug cost ($)					
Disitamab vedotin (60 mg)	533.40	426.72	640.08	Gamma	Yaozh (2025)
Toripalimab (80 mg)	114.10	91.28	136.92	Gamma	Yaozh (2025)
Gemcitabine (200 mg)	1.12	0.90	1.35	Gamma	Yaozh (2025)
Carboplatin (100 mg)	4.80	3.84	6.95	Gamma	Yaozh (2025)
Cisplatin (10 mg)	1.06	0.85	1.27	Gamma	Yaozh (2025)
Incidence of grade ≥3 AEs in DV + T group (%)					
Neutrophil count decreased	5.8	4.6	7.0	Beta	Sheng et al. (2025)
White-cell count decreased	3.7	3.0	4.4	Beta	Sheng et al. (2025)
Anemia	5.3	4.2	6.4	Beta	Sheng et al. (2025)
Platelet count decreased	0.8	0.6	1.0	Beta	Sheng et al. (2025)
Hypokalemia	5.8	4.6	7.0	Beta	Sheng et al. (2025)
Hypoesthesia	5.3	4.2	6.4	Beta	Sheng et al. (2025)
Lymphocyte count decreased	4.9	3.9	5.9	Beta	Sheng et al. (2025)
Incidence of grade ≥3 AEs in chemotherapy group (%)					
Neutrophil count decreased	61.7	49.4	74.0	Beta	Sheng et al. (2025)
White-cell count decreased	47.7	38.2	57.2	Beta	Sheng et al. (2025)
Anemia	45.5	36.4	54.6	Beta	Sheng et al. (2025)
Platelet count decreased	45.9	36.7	55.1	Beta	Sheng et al. (2025)
Hypokalemia	0.9	0.7	1.1	Beta	Sheng et al. (2025)
Hypoesthesia	0	0	0	Beta	Sheng et al. (2025)
Lymphocyte count decreased	11.7	9.4	14.0	Beta	Sheng et al. (2025)
Cost of adverse event ($)					
Neutrophil count decreased	119.15	95.32	142.98	Gamma	You et al. (2024)
White-cell count decreased	117.42	93.94	140.90	Gamma	You et al. (2024)
Anemia	143.74	115.00	172.49	Gamma	You et al. (2024)
Platelet count decreased	1,095.26	876.21	1,314.31	Gamma	You et al. (2024)
Hypokalemia	32.70	26.16	39.24	Gamma	Local Price
Hypoesthesia	9.06	7.25	10.87	Gamma	Local Price
Lymphocyte count decreased	56.00	44.80	67.20	Gamma	Local Price
Utility					
PFS	0.84	0.67	1.00	Beta	You et al. (2024)
PD	0.80	0.64	0.96	Beta	You et al. (2024)
Disutility					
Neutrophil count decreased	0.20	0.16	0.24	Beta	Shao et al. (2023)
White-cell decreased	0.20	0.16	0.24	Beta	Shao et al. (2023)
Anemia	0.07	0.06	0.08	Beta	Shao et al. (2023)
Platelet count decreased	0.19	0.15	0.23	Beta	Shao et al. (2023)
Hypokalemia	0.03	0.02	0.04	Beta	Assumption
Hypoesthesia	0.33	0.26	0.40	Beta	Ying and Fu (2026)
Lymphocyte count decreased	0.08	0.06	0.10	Beta	Assumption
Other					
Routine follow-up per cycle	89.0	71.2	106.8	Gamma	Zhang et al. (2021)
BSC care per cycle	220.1	176.1	264.1	Gamma	Zhang et al. (2021)
Tests per cycle	430.7	344.6	516.9	Gamma	Liu et al. (2023)
Terminal care	1,547.8	1,238.2	1857.3	Gamma	Liu et al. (2023)
Discount rate	0.045	0	0.05	Uniform	Wu and Liu (2025)
Body weight (kg)	65	52	78	Normal	You et al. (2024)
Body surface area (m^2^)	1.72	1.38	2.06	Normal	You et al. (2024)
Creatinine clearance rate (mL/min)	70	56	84	Gamma	You et al. (2024)

DV + T, disitamab vedotin plus toripalimab; AE, adverse event; BSC, the best supportive care; PD, progressive disease; PFS, progression-free survival.

### Sensitivity analysis

Both one-way and probabilistic sensitivity analyses were conducted to evaluate model robustness. In the one-way analyses, parameters including cost, utility, adverse-event incidence, body weight, body surface area and creatinine clearance rate were individually varied by ±20% from their baseline values or within their 95% confidence intervals. The discount rate was tested across a plausible range of 0%–5% (Wu and Liu, 2025). The results, summarized in a tornado diagram, identify the parameters with the greatest influence on the ICER. Probabilistic sensitivity analysis was performed using 1,000 Monte Carlo simulations, in which all parameters were varied simultaneously according to predefined distributions. The probabilistic sensitivity analyses results are presented with an incremental cost-effectiveness scatter plot and a cost-effectiveness acceptability curve. Additionally, a price threshold analysis was carried out to determine the price reduction required for the DV + T regimen to become cost-effective relative to chemotherapy. Drug prices for both DV and T were systematically lowered in the model until the ICER reached the WTP threshold.

### Subgroup analysis

The RC48-C016 trial presented Kaplan-Meier curves for OS and PFS across various subgroups. To assess the cost-effectiveness of DV + T across different patient populations, subgroup-specific ICERs were calculated based on these survival curves. Using the same digitization and reconstruction methods as for the overall population survival curves (Supplementary Figures S5 and S6), we extracted subgroup-specific survival data and calculated the corresponding ICERs. In these analyses, only the subgroup-specific survival curves were changed, while all other parameters remained consistent with those from the overall population. The subgroup analyses included stratification by the presence or absence of visceral metastases, eligibility for cisplatin-based chemotherapy, and HER2 IHC expression levels (1+ versus 2+/3+).

### Scenario analysis

We performed a scenario analysis to assess the robustness of the model outcomes under varying assumptions and clinical conditions. In scenario 1 and 2, the model time horizon was adjusted to 5 and 10 years while maintaining unchanged treatment protocols. In scenario 3 to 5, price reductions of 50%, 60%, and 70% for DV + T were examined to assess the impact of potential market or policy changes on cost-effectiveness. In immuno-oncology therapy, the treatment effect of immune agents often exhibits a delayed effect, leading to a plateau in the tail of the survival curve. Standard parametric models may not adequately capture such patterns. To assess this structural uncertainty, we applied the Royston-Parmar (R-P) spline model in Scenario 6. The best-fitting model was selected based on AIC and log-likelihood. Model selection and the best-fitting models are shown in Supplementary Tables S3, S4 and Supplementary Figure S7.

## Results

### Base case analysis

The results of this study are presented as total cost, quality-adjusted life years (QALYs), and incremental cost-effectiveness ratio (ICER) (Table 3). Compared with chemotherapy (1.43 QALYs at a cost of $28,300.87), the DV + T regimen yielded higher health outcomes (2.44 QALYs) but at a substantially higher total cost ($98,514.95). The incremental effectiveness and incremental cost of DV + T were 1.01 QALYs and $70,214.09, respectively, resulting in an ICER of $69,575.31 per QALY gained versus chemotherapy. At the WTP threshold in China ($20,167.02 per QALY), DV + T is not cost-effective compared with chemotherapy.

**TABLE 3:** Cost and outcomes of the cost-effectiveness analyses.

Regimen	DV + T	Chemotherapy	Incremental
Total cost, $	98,514.95	28,300.87	70,214.09
Total QALYs	2.44	1.43	1.01
ICER, per QALY	​	​	69,575.31

DV + T, disitamab vedotin plus toripalimab; ICER, incremental cost-effectiveness ratio; QALY, quality-adjusted life year.

### Sensitivity analysis

**FIGURE 2:**
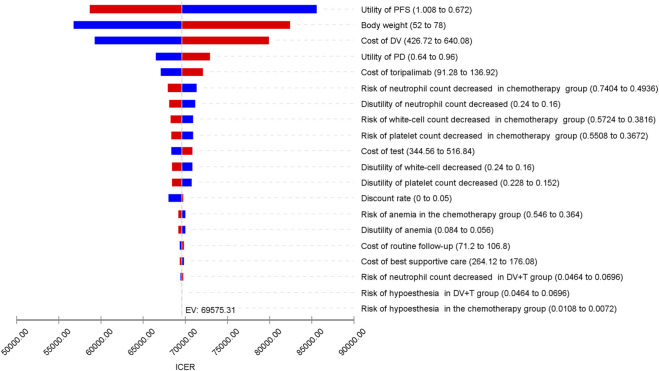
One-way sensitivity analyses of DV + T in comparison to chemotherapy. DV + T, disitamab vedotin plus toripalimab; ICER, incremental cost-effectiveness ratio; PFS, progression-free survival; PD, progressive disease.

**FIGURE 3:**
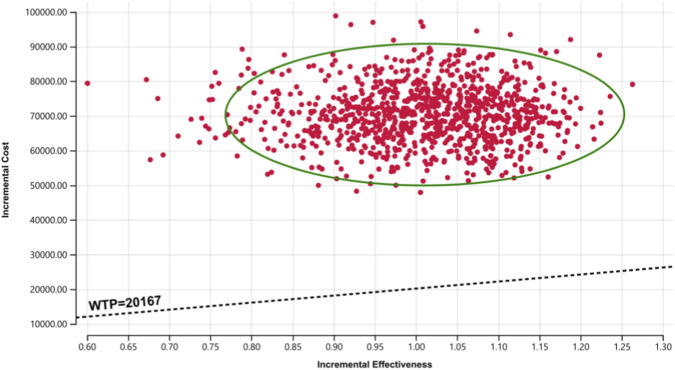
Incremental cost-effectiveness scatter plot of DV + T and chemotherapy. DV + T, disitamab vedotin plus toripalimab.

**FIGURE 4:**
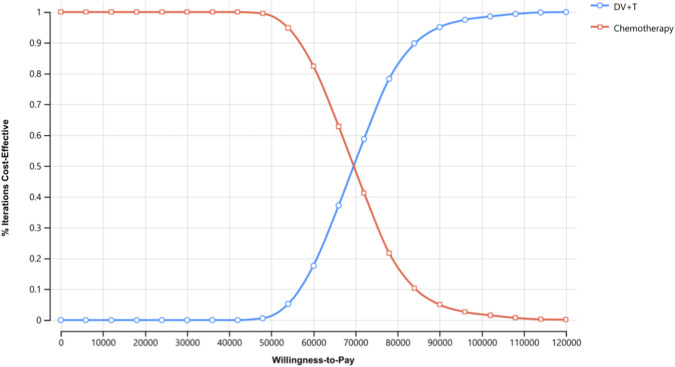
The cost-effectiveness acceptability curve for DV + T versus chemotherapy. DV + T, disitamab vedotin plus toripalimab; QALY, quality-adjusted life year.

The one-way sensitivity analysis results are presented in a tornado diagram (Figure 2). The dominant drivers of model uncertainty were the utility of PFS, body weight, the price of DV, the utility of PD, and the price of T. Even when these parameters were varied across their plausible ranges, the resulting ICER consistently exceeded the WTP threshold. This indicates that the model outcomes remained robust to variations in these inputs. The probabilistic sensitivity analysis is illustrated as cost-effectiveness scatter plot (Figure 3) and acceptability curve (Figure 4). At the prespecified WTP threshold, the probability that DV + T is cost-effective compared to chemotherapy was 0%.

### Subgroup analysis

Across all analyzed subgroups, the ICERs for DV + T consistently exceeded $20,167.02 per QALY with a 0% probability of cost-effectiveness, indicating that it is not a cost-effective option versus chemotherapy (Table 4). The ICERs were numerically lower in patients without visceral metastases and those with HER2 IHC 1+ expression. Notably, the ICER was substantially lower in patients ineligible to receive cisplatin ($61,264.33 per QALY) than in those eligible for cisplatin-based chemotherapy ($245,983.12 per QALY).

**TABLE 4:** Results of subgroup analyses.

Subgroup	Regimen	No. of patients	No. of events	Total cost ($)	Total QALYs	ICER (per QALY)
Visceral metastases	DV + T	124	72	72,637.18	1.61	100,299.67
	Chemo	126	87	19,713.49	1.08	
Without visceral metastases	DV + T	119	54	117,513.20	3.39	51,721.71
	Chemo	115	62	25,636.45	1.61	
Eligible to receive cisplatin	DV + T	127	70	78,482.44	1.82	245,983.12
	Chemo	128	76	31,295.50	1.63	
Ineligible to receive cisplatin	DV + T	116	56	104,373.89	2.51	61,264.33
	Chemo	113	73	24,560.34	1.21	
HER2 IHC 1+	DV + T	55	33	74,231.39	1.95	49,213.22
	Chemo	53	31	18,377.35	0.82	
HER2 IHC 2+/3+	DV + T	188	93	92,974.75	2.28	87,081.04
	Chemo	188	118	29,859.09	1.56	

DV + T, disitamab vedotin plus toripalimab; Chemo, chemotherapy; ICER, incremental cost-effectiveness ratio; QALY, quality-adjusted life year.

### Scenario analysis

The results of the scenario analysis are shown in Table 5. In both scenario 1 and 2, extending the model’s time horizon was associated with a gradual reduction in the ICER for DV + T versus chemotherapy. In Scenarios 3 to 5, the ICER for DV + T decreased as the price of DV + T declined. To reach comparable cost-effectiveness, the prices of DV and T were reduced to $122.42 and $26.19, respectively, which corresponded to 22.95% of their original prices. In Scenario 6, using the RP spline model, the calculated ICER was $67,084.26. At the WTP threshold, the probability of DV + T being cost-effective was 0%.

**TABLE 5:** Results of scenario analyses.

Scenario		Cost		QALY		ICER (per QALY)	Probability of cost-effectiveness
		DV + T	Chemotherapy	DV + T	Chemotherapy		
1	Time horizon = 5 years	82,608.33	58,889.27	1.93	1.15	75,351.06	0
2	Time horizon = 10 years	94,381.06	27,111.70	2.31	1.36	70,901.36	0
3	50% Price reduction of DV + T	66,160.78	28,300.87	2.44	1.43	37,515.48	0
4	60% Price reduction of DV + T	59,689.94	28,300.87	2.44	1.43	31,103.51	0.1%
5	70% Price reduction of DV + T	53,219.11	28,300.87	2.44	1.43	24,691.55	6.7%
6	RP spline model	98,653.82	27,214.73	2.45	1.39	67,084.26	0

DV + T, disitamab vedotin plus toripalimab; ICER, incremental cost-effectiveness ratio; QALY, quality-adjusted life year; PFS, progression-free survival; PD, progressive disease.

## Discussion

Our analysis demonstrated that DV + T was not a cost-effective first-line option for patients with HER2-expressing advanced UC in China. Compared with chemotherapy, DV + T cost an additional $69,575.31 per QALY, substantially exceeding the pre-specified WTP threshold of $20,167.02 per QALY. One-way sensitivity analyses identified the high acquisition costs of DV and T as the primary drivers of this lack of cost-effectiveness. Therefore, we further estimated the price reductions required for DV + T to reach the WTP threshold. DV + T became cost-effective only when the prices of both drugs were reduced to 22.95% of their original levels, corresponding to $122.42 for DV and $26.19 for T.

To date, no pharmacoeconomic study has specifically focused on HER2-expressing advanced UC. However, from the perspective of the Chinese healthcare system, three economic evaluations have assessed ADC plus ICI regimens for advanced UC using different comparators (Wu and Liu, 2025; Zhu et al., 2025). Two studies compared enfortumab vedotin plus pembrolizumab (EV + P) with chemotherapy. One reported an ICER of $232,256.16 per QALY, far exceeding the WTP threshold of $38,133, with the high cost of EV and P identified as the primary driver. The other yielded an ICER of $267,491 per QALY, also above the WTP threshold of $35,173, where the price of EV was the main contributing factor (Zhu et al., 2025). A third study, comparing EV + P with nivolumab plus gemcitabine-cisplatin, reported an ICER of $351,960.68 per QALY, exceeding the corresponding threshold of $40,451.64, with the cost of EV and P similarly identified as the key determinant (Ying and Fu, 2026). Collectively, these studies consistently demonstrated that ADC plus ICI regimen was not cost-effective as first-line treatment for advanced UC in China, a finding consistent with our conclusions.

Since 2016, China’s National Drug Price Negotiation (NDPN) policy has significantly reduced the prices of pharmaceuticals, especially innovative anticancer drugs. This has alleviated the financial burden on patients, insurance funds, and the healthcare system, while simultaneously enhancing drug accessibility (Cai L. et al., 2022). The high cost of the EV + P regimen and its exclusion from China’s National Reimbursement Drug List (NRDL) are the primary barriers to its clinical accessibility. Similarly, although DV and T have been included in NRDL through price negotiation, their use as first-line treatment for HER2-expressing advanced UC is not currently reimbursed. Moreover, no patient assistance programs (PAPs) or charitable drug donation programs are available for these drugs, which further limits their cost-effectiveness. To achieve an acceptable ICER, further price reductions or indication-specific negotiation would be required. Given the favorable efficacy data observed in our study, policymakers may consider expanding the current reimbursement scope to include this indication, or alternatively, manufacturer-sponsored drug donation programs, could be established to reduce out-of-pocket costs for eligible patients. Such measures would not only improve the cost-effectiveness profile but also enhance equitable access to this therapy for patients with HER2-expressing advanced UC.

We further conducted exploratory subgroup cost-effectiveness analyses. The cisplatin-ineligible and without visceral metastases subgroups appeared to exhibit a more favorable cost-effectiveness trend, although their ICER values still exceeded the WTP threshold. These exploratory findings suggest that such populations could be prioritized for clinical implementation if future price negotiations succeed. Furthermore, different HER2 expression levels also influenced the outcomes: the HER2 2+/3+ subgroup achieved slightly higher QALYs than the HER2 1+ subgroup, yet accompanied by a correspondingly higher ICER. Given the relatively small sample size in the HER2 1+ subgroup, this conclusion should be interpreted with caution. All subgroup findings are exploratory and warrant confirmation in larger prospective studies.

Our study has several notable strengths. First, to our knowledge, this is the first cost-effectiveness analysis of DV + T as a first-line treatment for advanced UC. This pioneering assessment provides crucial evidence to inform healthcare policy and clinical decision-making, particularly in resource-constrained settings like China. Second, our subgroup analyses offer insights for clinical prioritization. The economic trends observed in key subgroups, such as cisplatin-ineligible patients and those with visceral metastases, identify populations that may derive greater value from this regimen. These findings can help guide the efficient allocation of limited healthcare resources. Third, the robustness of our conclusions is strengthened by extensive scenario and sensitivity analyses. We systematically tested key model assumptions and parameters, including time horizons, health utility values, and drug pricing scenarios. The consistency of the findings across these analyses enhances the reliability of our model and its policy implications.

Several limitations should be noted. First, given the lack of long-term survival data from the RC48-C016 trial, extrapolation of survival curves over a 15-year horizon using the best-fitting parametric distribution may introduce uncertainty. Second, the model assumed that all patients received best supportive care after progression due to a lack of data on subsequent therapies; this may not fully reflect real-world scenarios where some patients receive further active treatment. Third, the analysis considered only grade ≥3 adverse events with an incidence exceeding 5%, omitting potential costs and utility impacts of lower-grade events. Lastly, formal external validation using long-term real-world data is not yet feasible because the RC48-C016 trial was only recently completed. These limitations should be considered when interpreting the findings.

## Conclusion

In conclusion, based on the available data, DV + T is not expected to be a cost-effective option for the treatment of previously untreated HER2-expressing advanced UC patients from the perspectives of payers in China at the appropriate WTP threshold. These results have the potential to guide clinical decision-making regarding the management of advanced UC patients or the establishment of appropriate pricing and medical reimbursement policies for these drugs. However, these results demonstrate that DV + T treatment does afford patients significant clinical benefits while simultaneously imposing a heavy economic burden. Efforts to reduce the cost of DV + T may be highly effective as a means of improving the accessibility of this innovative therapeutic regimen in the clinic.

## Data Availability

The data analyzed in this study is subject to the following licenses/restrictions: The datasets used in this study were obtained from the published RC48-C016 clinical trial. The original trial data are owned by the trial sponsor and are not publicly available due to privacy and confidentiality restrictions. Access to these data may be granted upon reasonable request to the trial sponsor. Requests to access these datasets should be directed to Xiaolin Yun, yunxiaolin00@126.com.
