# Supplementary material for: Disitamab vedotin plus toripalimab as a first-line treatment for HER2-expressing advanced urothelial cancer: a cost-effectiveness analysis from China based on the RC48-C016 trial
Source: Front Pharmacol. 2026 Jul 17;17:1824661. doi: 10.3389/fphar.2026.1824661 (PMC13423685; doi:10.3389/fphar.2026.1824661)
Supplement: Supplementary file 1 [file Supplementaryfile1.docx]

**Supplementary Materials**

**Supplementary Table 1.** CHEERS Checklist 2022

**Supplementary Table 2.** Summary of statistical goodness-of-fit of Kaplan-Meier curves

**Supplementary Figure 1.** Fitting and extrapolation of PFS curves for disitamab vedotin plus toripalimab

**Supplementary Figure 2.** Fitting and extrapolation of OS curves for disitamab vedotin plus toripalimab

**Supplementary Figure 3.** Fitting and extrapolaion of PFS curves for chemotherapy

**Supplementary Figure 4.** Fitting and extrapolation of OS curves for chemotherapy

**Supplementary Figure 5.** Reconstructed PFS curves for subgroups.

**Supplementary Figure 6.** Reconstructed OS curves for subgroups.

**Supplementary Table 3.** Summary of Royston-Parmar spline models fitted to the Kaplan-Meier curves

**Supplementary Table 4.** Parameter estimates of the best-fitting Royston-Parmar spline models

**Supplementary Figure 7.** Fitted survival curves from the best-fitting Royston-Parmar spline models.

**Supplementary Table 1.** The CHEERS 2022 checklist.

| **Section/item** | **No.** | **Item** | **Reported** |
| --- | --- | --- | --- |
| **Title and abstract** | | | |
| Title | 1 | Identify the study as an economic evaluation and specify the interventions being compared. | Title |
| Abstract | 2 | Provide a structured summary that highlights context, key methods, results, and alternative analyses. | Abstract |
| **Introduction** | | | |
| Background and objectives | 3 | Give the context for the study, the study question, and its practical relevance for decision making in policy or practice. | Introduction |
| **Methods** | | | |
| Health economic analysis plan | 4 | Indicate whether a health economic analysis plan was developed and where available. | Not applicable |
| Study population | 5 | Describe characteristics of the study population (such as age range, demographics, socioeconomic, or clinical characteristics). | Methods: Clinical information |
| Setting and location | 6 | Provide relevant contextual information that may influence findings. | Methods: Clinical information |
| Comparators | 7 | Describe the interventions or strategies being compared and why chosen. | Methods: Constructing the model |
| Perspective | 8 | State the perspective(s) adopted by the study and why chosen. | Methods: Constructing the model |
| Time horizon | 9 | State the time horizon for the study and why appropriate. | Methods: Constructing the model |
| Discount rate | 10 | Report the discount rate(s) and reason chosen. | Methods: Constructing the model |
| Selection of outcomes | 11 | Describe what outcomes were used as the measure(s) of benefit(s) and harm(s) | Methods: Constructing the model |
| Measurement of outcomes | 12 | Describe how outcomes used to capture benefit(s) and harm(s) were measured. | Methods: Constructing the model |
| Valuation of outcomes | 13 | Describe the population and methods used to measure and value outcomes. | Methods: Clinical information, Constructing the model |
| Measurement and valuation of resources and costs | 14 | Describe how costs were valued. | Methods: Costs and utility |
| Currency, price date, and conversion | 15 | Report the dates of the estimated resource quantities and unit costs, plus the currency and year of conversion. | Methods: Costs and utility |
| Rationale and description of model | 16 | If modelling is used, describe in detail and why used. Report if the model is publicly available and where it can be accessed. | Methods: Constructing the model, Clinical information |
| Analytics and assumptions | 17 | Describe any methods for analysing or statistically transforming data, any extrapolation methods, and approaches for validating any model used. | Methods: Constructing the model, Supplementary Table 1-4 and Figure 7 |
| Characterizing heterogeneity | 18 | Describe any methods used for estimating how the results of the study vary for subgroups. | Methods: Subgroup analysis |
| Characterizing distributional effects | 19 | Describe how impacts are distributed across different individuals or adjustments made to reflect priority populations. | Not applicable |
| Characterizing uncertainty | 20 | Describe methods to characterise any sources of uncertainty in the analysis. | Methods: Sensitivity analysis, Scenario analysis |
| Approach to engagement with patients and others affected by the study | 21 | Describe any approaches to engage patients or service recipients, the general public, communities, or stakeholders (such as clinicians or payers) in the design of the study. | Not applicable |
| **Results** | | | |
| Study parameters | 22 | Report all analytic inputs (such as values, ranges, references) including uncertainty or distributional assumptions. | Table 2 and 5, Supplementary Table 2-4 |
| Summary of main results | 23 | Report the mean values for the main categories of costs and outcomes of interest and summarise them in the most appropriate overall measure. | Results: Base case analysis, Table 3-5 |
| Effect of uncertainty | 24 | Describe how uncertainty about analytic judgments, inputs, or projections affect findings. Report the effect of choice of discount rate and time horizon, if applicable. | Results:  Sensitivity analysis, Scenario analysis, Table 5 |
| Effect of engagement with patients and others affected by the study | 25 | Report on any difference patient/service recipient, general public, community, or stakeholder involvement made to the approach or findings of the study. | Not  applicable |
| **Discussion** | | | |
| Study findings, limitations, generalizability, and current knowledge | 26 | Report key findings, limitations, ethical or equity considerations not captured, and how these could affect patients, policy, or practice. | Discussion |
| **Other** | | | |
| Source of funding | 27 | Describe how the study was funded and any role of the funder in the identification, design, conduct, and reporting of the analysis | Funding |
| Conflicts of interest | 28 | Report authors conflicts of interest according to journal or International Committee of Medical Journal Editors requirements. | Conflict of Interest |

**Supplementary Table 2.** Summary of statistical goodness-of-fit of Kaplan-Meier curve.

|  | **DV+T** | | **Chemotherapy** | |
| --- | --- | --- | --- | --- |
|  | AIC | BIC | AIC | BIC |
| **OS curve** | | | | |
| **Exponential** | 781.2099 | 784.7030 | 1003.9274 | 1007.412 |
| **Weibull** | 770.4787 | 777.4648 | 995.7027 | 1002.672 |
| **gamma** | 770.2147 | 777.2008 | 995.6034 | 1002.573 |
| **Log-logistic** | **770.1622** | **777.1483** | **994.9921** | **1001.962** |
| **Log-normal** | 773.1097 | 780.0958 | 1008.4924 | 1015.462 |
| **Gompertz** | 774.7238 | 781.7099 | 1000.5211 | 1007.491 |
| **Generalized gamma** | 772.1427 | 782.6219 | 997.5466 | 1008.001 |
| **PFS curve** | | | | |
| **Exponential** | 1014.322 | 1017.815 | 961.1177 | 964.6025 |
| **Weibull** | 1006.706 | 1013.693 | **934.4794** | **941.4490** |
| **gamma** | 1005.516 | 1012.502 | 938.6553 | 945.6249 |
| **Log-logistic** | **1004.627** | **1011.613** | 945.6350 | 952.6046 |
| **Log-normal** | 1009.021 | 1016.007 | 997.0324 | 1004.0020 |
| **Gompertz** | 1012.321 | 1019.307 | 945.2376 | 952.2072 |
| **Generalized gamma** | 1006.716 | 1017.195 | 936.2380 | 946.6924 |

AIC, Akaike information criterion; BIC, Bayesian information criterion; DV+T, disitamab vedotin plus toripalimab; OS, overall survival; PFS, progression-free survival.


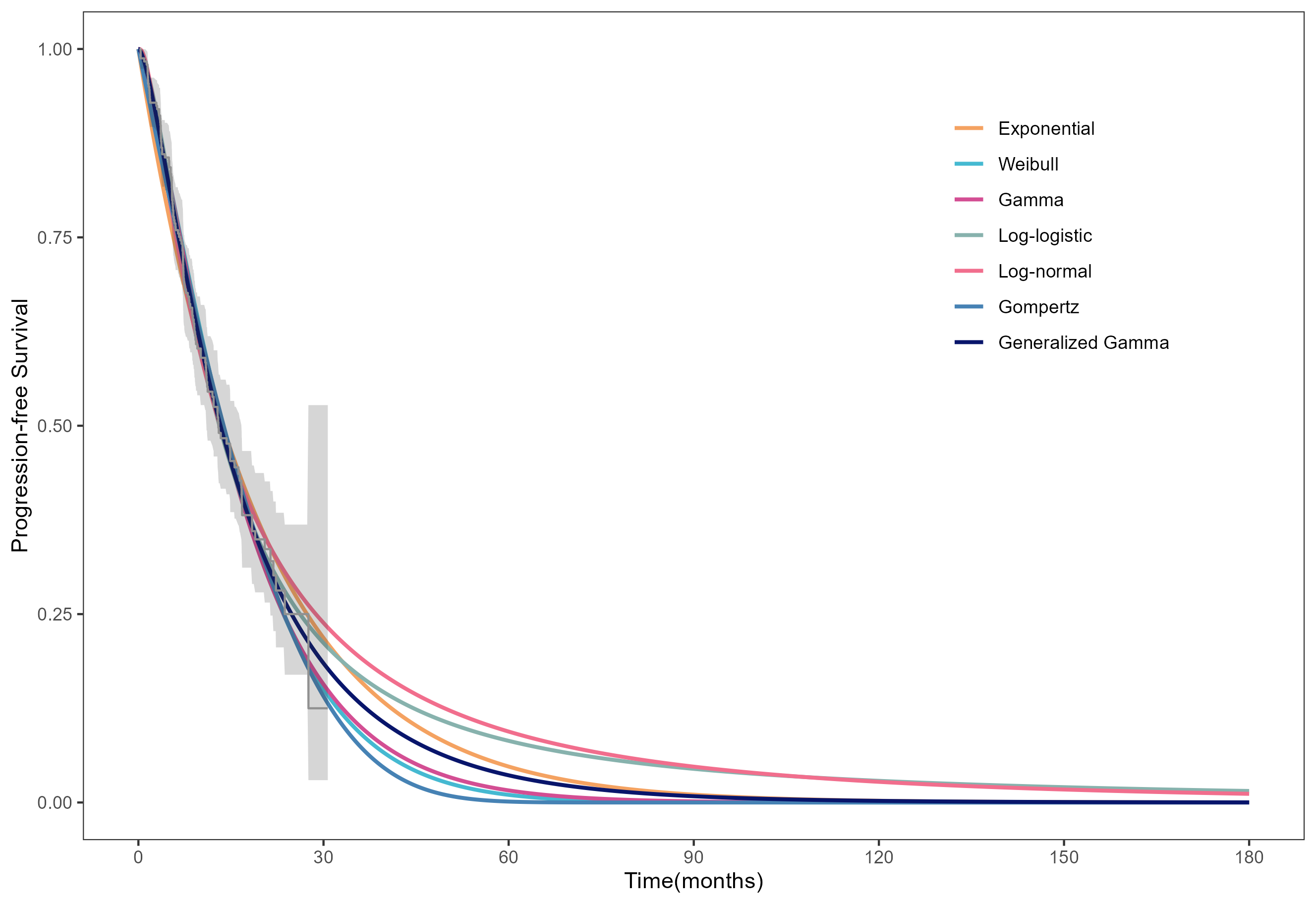


**Supplementary Figure 1.** Fitting and extrapolation of PFS curves for disitamab vedotin plus toripalimab


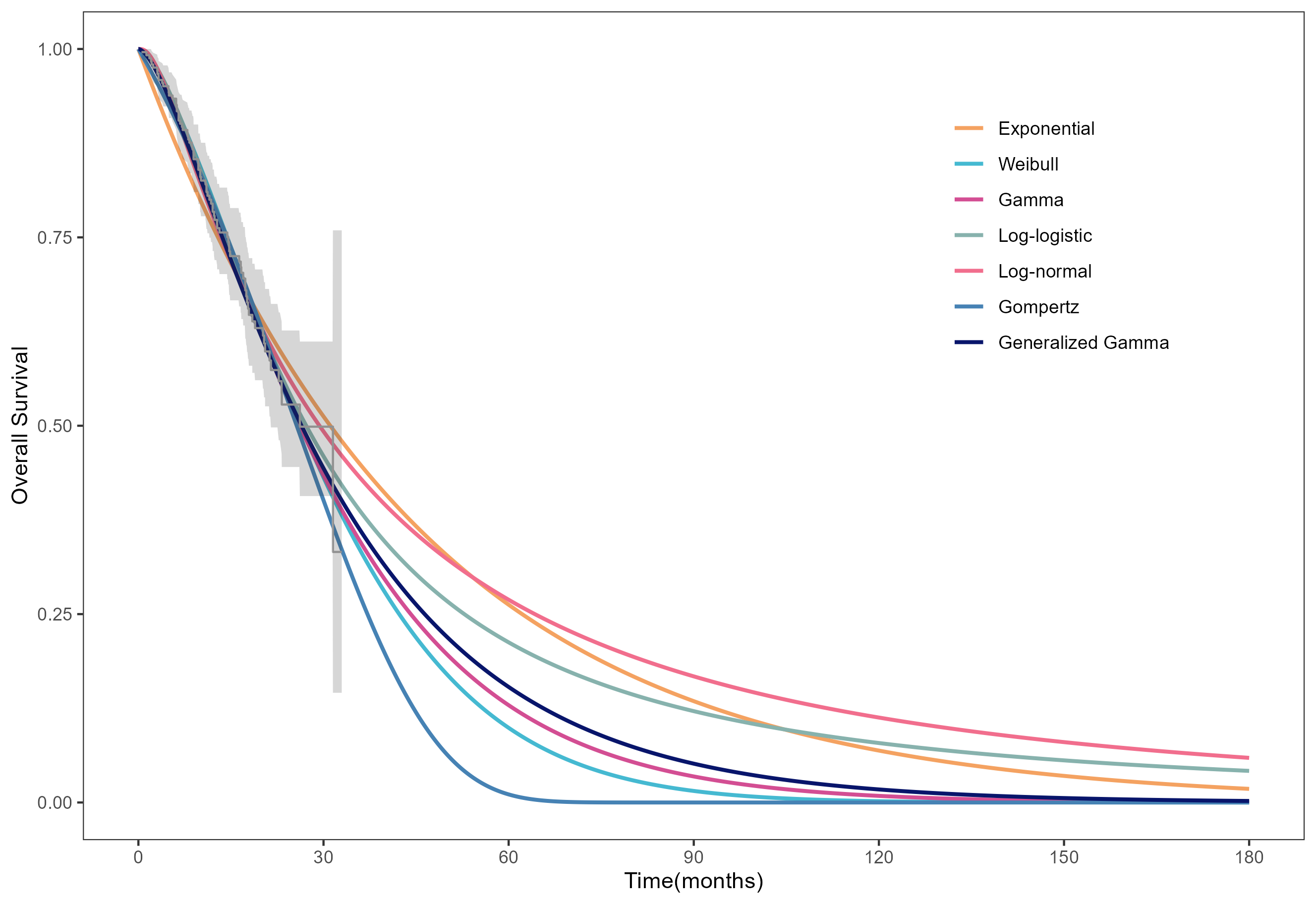


**Supplementary Figure 2.** Fitting and extrapolation of OS curves for disitamab vedotin plus toripalimab


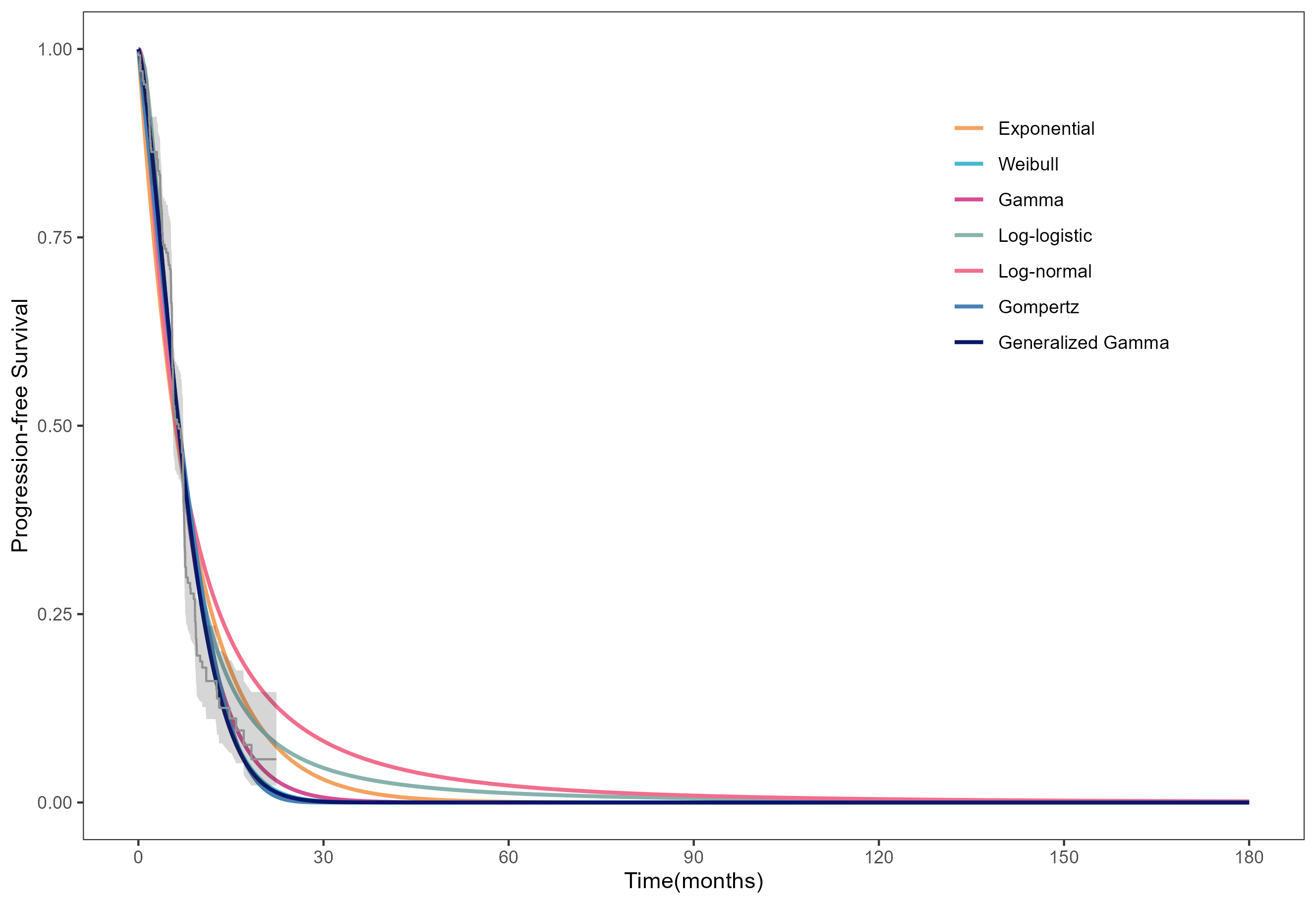


**Supplementary Figure 3.** Fitting and extrapolation of PFS curves for chemotherapy


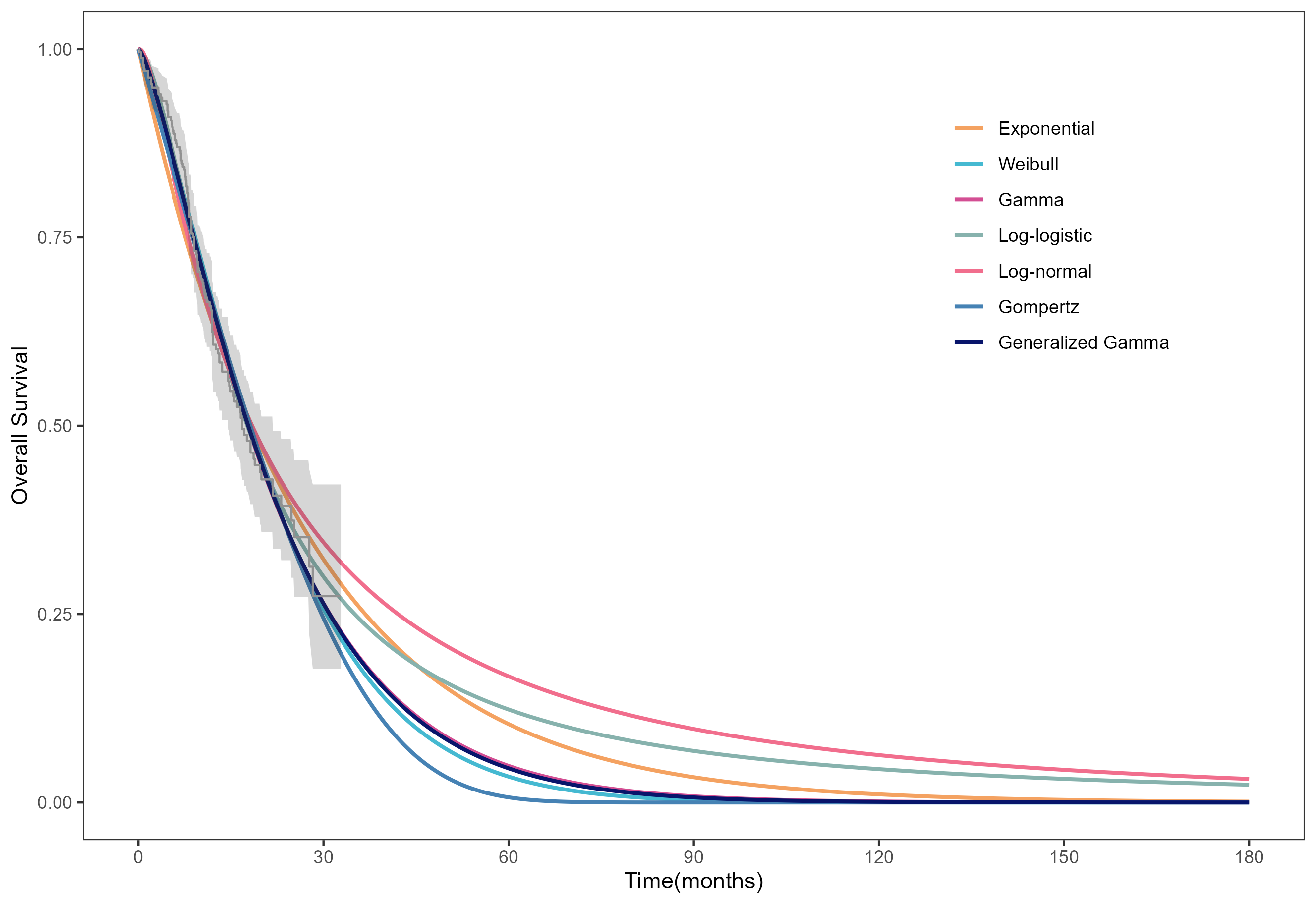


**Supplementary Figure 4.** Fitting and extrapolation of OS curves for chemotherapy


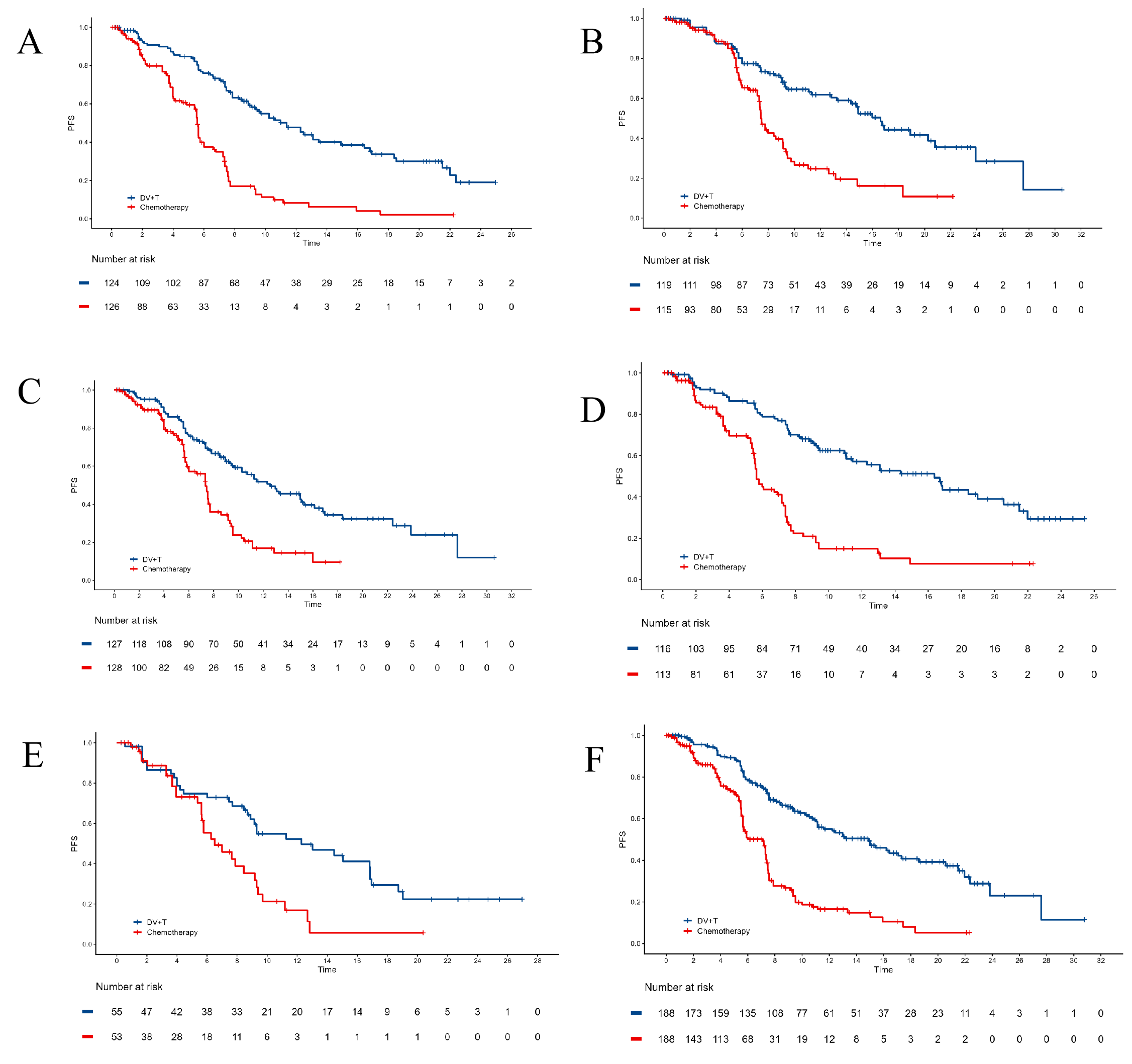


**Supplementary Figure 5.** Reconstructed PFS curves for subgroups. (A) Reconstructed PFS curves for subgroups with visceral metastases, (B) reconstructed PFS curves for subgroups with visceral metastases, (C) reconstructed PFS curves for subgroups eligible to receive cisplatin, (D) reconstructed PFS curves for subgroups ineligible to receive cisplatin, (E) reconstructed PFS curves for subgroups with HER2 IHC 1+, (F) reconstructed PFS curves for subgroups with HER2 IHC 2+/3+


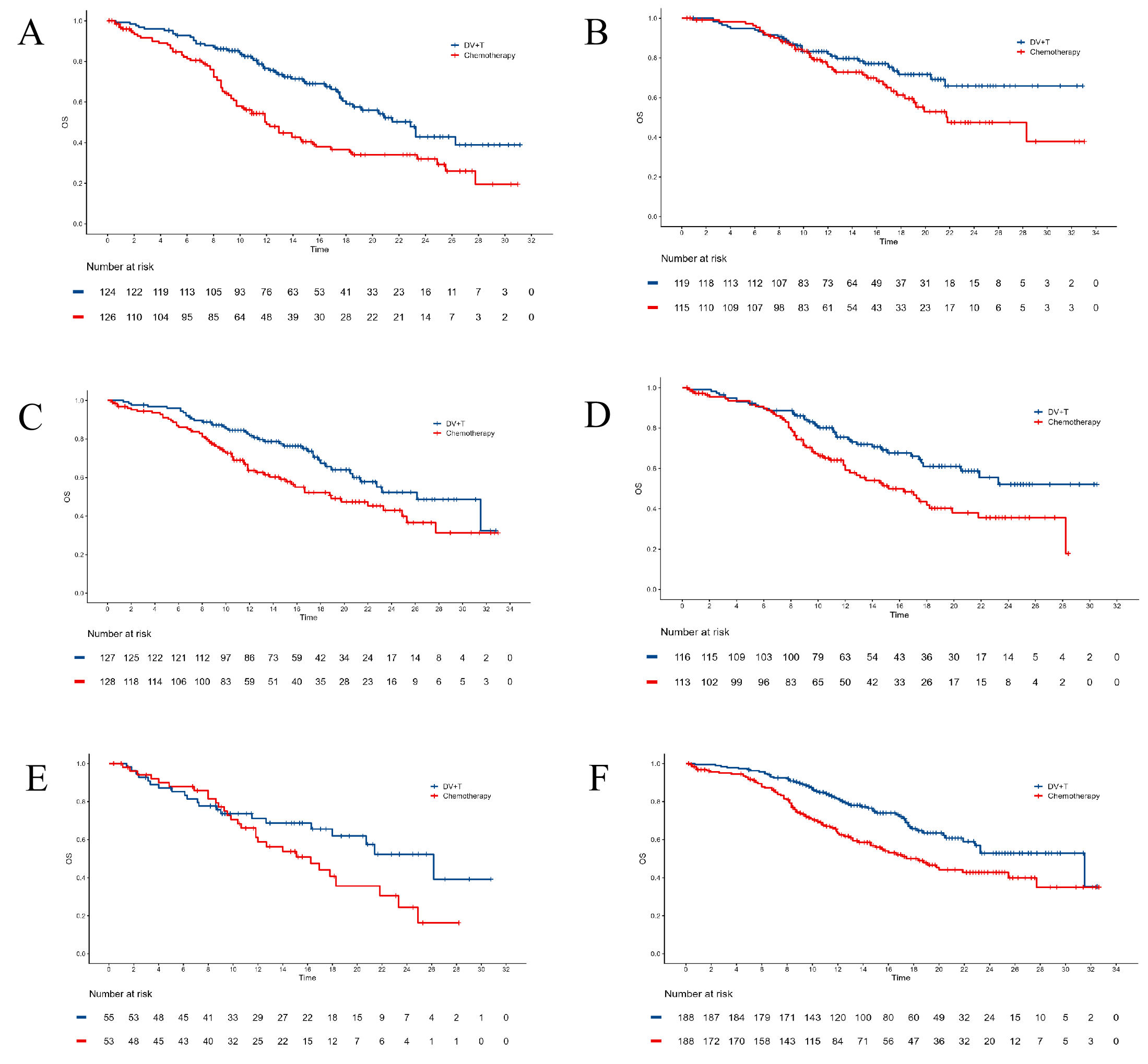


**Supplementary Figure 6.** Reconstructed OS curves for subgroups. (A) Reconstructed OS curves for subgroups with visceral metastases, (B) reconstructed OS curves for subgroups with visceral metastases, (C) reconstructed OS curves for subgroups eligible to receive cisplatin, (D) reconstructed OS curves for subgroups ineligible to receive cisplatin, (E) reconstructed OS curves for subgroups with HER2 IHC 1+, (F) reconstructed OS curves for subgroups with HER2 IHC 2+/3+

**Supplementary Table 3.** Summary of Royston-Parmar spline models fitted to the Kaplan-Meier curves.

|  | Model | Parameters | AIC | LnL |
| --- | --- | --- | --- | --- |
| DVT-PFS | RP-hazard-1 | 2 | 169.93 | -82.96 |
|  | RP-hazard-2 | 3 | 169.78 | -81.89 |
|  | **RP-odds-1** | **2** | **167.52** | **-81.76** |
|  | RP-odds-2 | 3 | 169.48 | -81.74 |
|  | RP-normal-1 | 3 | 169.77 | -81.89 |
|  | RP-normal-2 | 4 | 170.87 | -81.44 |
| DVT-OS | RP-hazard-1 | 2 | 140.71 | -68.36 |
|  | RP-hazard-2 | 3 | 142.68 | -68.34 |
|  | **RP-odds-1** | **2** | **140.66** | **-68.33** |
|  | RP-odds-2 | 3 | 142.51 | -68.26 |
|  | RP-normal-1 | 3 | 142.56 | -68.28 |
|  | RP-normal-2 | 2 | 145.03 | -70.51 |
| Chemo-PFS | RP-hazard-1 | 6 | 175.63 | -81.81 |
|  | **RP-hazard-2** | **5** | **174.95** | **-82.48** |
|  | RP-odds-1 | 5 | 175.05 | -82.53 |
|  | RP-odds-2 | 6 | 175.70 | -81.85 |
|  | RP-normal-1 | 6 | 175.64 | -81.82 |
|  | RP-normal-2 | 5 | 175.57 | -82.78 |
| Chemo-OS | **RP-hazard-1** | **4** | **156.20** | **-74.10** |
|  | RP-hazard-2 | 7 | 157.32 | -71.66 |
|  | RP-odds-1 | 7 | 157.59 | -71.80 |
|  | RP-odds-2 | 4 | 156.87 | -74.44 |
|  | RP-normal-1 | 7 | 157.37 | -71.69 |
|  | RP-normal-2 | 6 | 158.64 | -73.32 |

AIC, Akaike information criterion; LnL, Log-likelihood

**Supplementary Table 4.** Parameter estimates of the best-fitting Royston-Parmar spline model.

|  | Model | Parameter | est |
| --- | --- | --- | --- |
| DVT-PFS | RP-odds-1 | Gamma 0 | -0.1376 |
|  |  | Gamma 1 | 1.5873 |
| DVT-OS | RP-odds-1 | Gamma 0 | -1.3400 |
|  |  | Gamma 1 | 1.6471 |
| Chemo-PFS | RP-hazard-2 | Gamma 0 | -2.6790 |
|  |  | Gamma 1 | 0.4707 |
|  |  | Gamma 2 | 1.1910 |
|  |  | Gamma 3 | -5.2288 |
|  |  | Gamma 4 | 4.6976 |
| Chemo-OS | RP-hazard-1 | Gamma 0 | -2.5497 |
|  |  | Gamma 1 | 0.7147 |
|  |  | Gamma 2 | -0.8107 |
|  |  | Gamma 3 | 1.0890 |


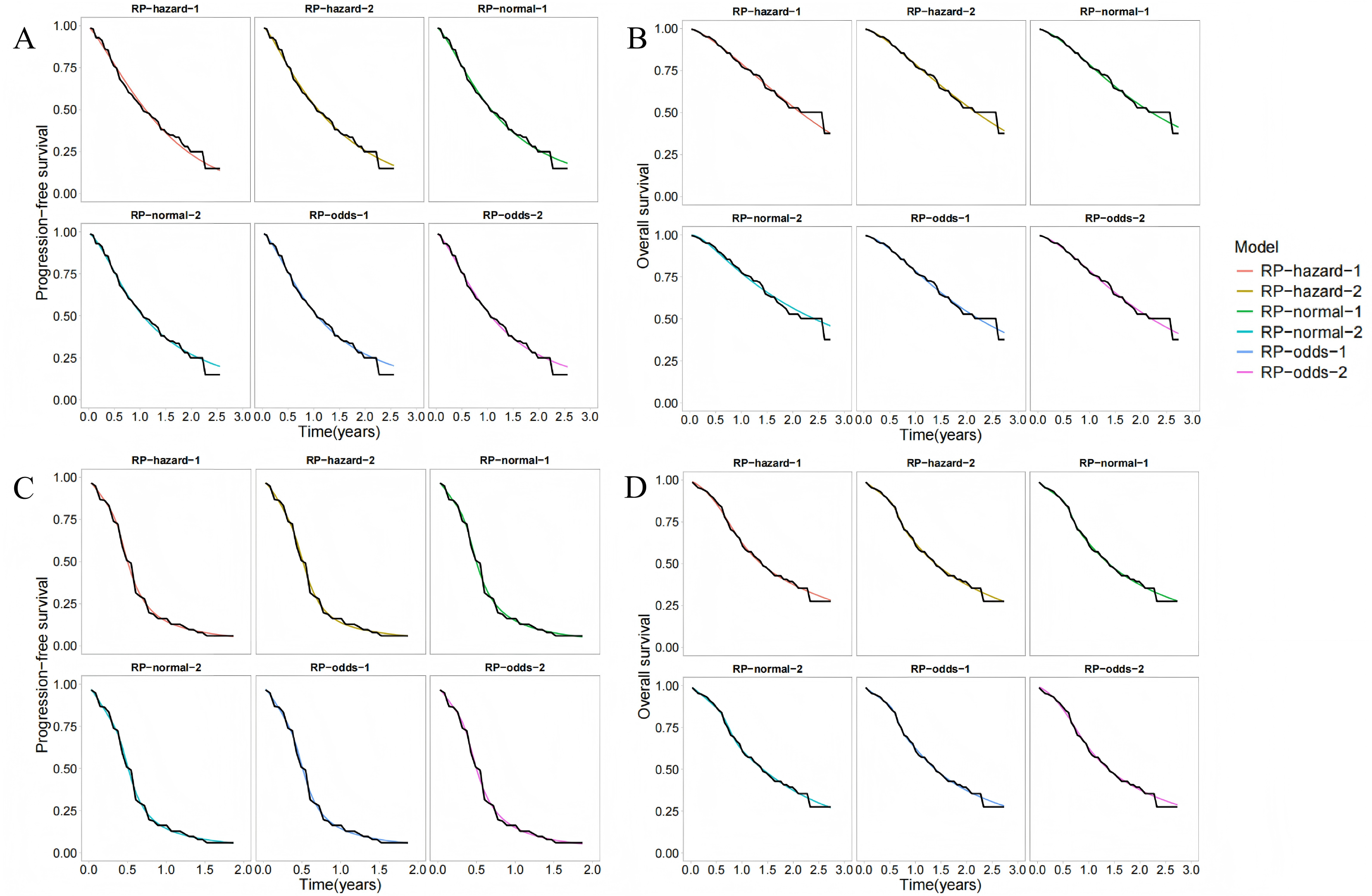


**Supplementary Figure 7.** Fitted survival curves from the best-fitting Royston-Parmar spline models. (A) PFS curves in the DV+T group, (B) OS curves in the DV+T group, (C) PFS curves in the chemotherapy group, (D) OS curves in the chemotherapy group.
